# Three-dimensional morphology and gene expression in the *Drosophila *blastoderm at cellular resolution I: data acquisition pipeline

**DOI:** 10.1186/gb-2006-7-12-r123

**Published:** 2006-12-21

**Authors:** Cris L Luengo Hendriks, Soile VE Keränen, Charless C Fowlkes, Lisa Simirenko, Gunther H Weber, Angela H DePace, Clara Henriquez, David W Kaszuba, Bernd Hamann, Michael B Eisen, Jitendra Malik, Damir Sudar, Mark D Biggin, David W Knowles

**Affiliations:** 1 Berkeley *Drosophila *Transcription Network Project, Life Sciences Division, Lawrence Berkeley National Laboratory, One Cyclotron Road, Berkeley, CA 94720, USA; 2 Berkeley *Drosophila *Transcription Network Project, Genomics Division, Lawrence Berkeley National Laboratory, One Cyclotron Road, Berkeley, CA 94720, USA; 3 Berkeley *Drosophila *Transcription Network Project, Department of Electrical Engineering and Computer Science, University of California, Berkeley, CA 94720, USA; 4 Berkeley *Drosophila *Transcription Network Project, Institute for Data Analysis and Visualization, University of California, Davis, CA 95616, USA

## Abstract

**Background:**

To model and thoroughly understand animal transcription networks, it is essential to derive accurate spatial and temporal descriptions of developing gene expression patterns with cellular resolution.

**Results:**

Here we describe a suite of methods that provide the first quantitative three-dimensional description of gene expression and morphology at cellular resolution in whole embryos. A database containing information derived from 1,282 embryos is released that describes the mRNA expression of 22 genes at multiple time points in the *Drosophila *blastoderm. We demonstrate that our methods are sufficiently accurate to detect previously undescribed features of morphology and gene expression. The cellular blastoderm is shown to have an intricate morphology of nuclear density patterns and apical/basal displacements that correlate with later well-known morphological features. Pair rule gene expression stripes, generally considered to specify patterning only along the anterior/posterior body axis, are shown to have complex changes in stripe location, stripe curvature, and expression level along the dorsal/ventral axis. Pair rule genes are also found to not always maintain the same register to each other.

**Conclusion:**

The application of these quantitative methods to other developmental systems will likely reveal many other previously unknown features and provide a more rigorous understanding of developmental regulatory networks.

## Abstract 2

A suite of methods that provide the first quantitative three-dimensional description of gene expression and morphology with cellular resolution in whole *Drosophila *embryos is described.

## Background

Animal embryos can be thought of as dynamic three-dimensional arrays of cells expressing gene products in intricate spatial and temporal patterns that determine cellular differentiation and morphogenesis. Although developmental biologists most commonly analyze gene expression and morphology by visual inspection of photographic images, it has been increasingly recognized that a rigorous understanding of developmental processes requires automated methods that quantitatively record and analyze these phenomenally complex spatio-temporal patterns at cellular resolution. Different imaging and image analysis methods have been used to provide one-, two-, or three-dimensional descriptions of parts or all of a developing animal at various levels of detail (for example, [1-9]). Yet, none of these experiments have described the morphology and gene expression of a complete embryo at cellular resolution.

The Berkeley *Drosophila *Transcription Network Project (BDTNP) [10] has initiated an interdisciplinary analysis of the transcription network in the early *Drosophila *embryo [11,12]. The project's goals are to develop techniques for deciphering the transcriptional regulatory information encoded in the genome and quantitatively model how regulatory interactions within the network generate spatial and temporal patterns of gene expression. Multiple system-wide datasets are being generated, including information on the *in vivo *and *in vitro *DNA binding specificities of the trans-acting factors that control the network. In this paper, we introduce a complementary dataset that describes the expression patterns of key transcription factors and a subset of their target genes in three dimensions for the whole embryo at cellular resolution, together with the methods we have developed to generate and analyze these data. By comparing the patterns of expression of the trans-regulators to those of their presumptive targets, we hope to provide evidence for the regulatory relationships within the network and allow modeling of how gene expression patterns develop.

The *Drosophila *blastoderm was chosen as the model to study as it is one of the best characterized animal regulatory networks [13-16]. Two and a half hours after fertilization, the embryo is a syncytium of around 6,000 nuclei, which then become cellularized by an enveloping membrane during developmental stage 5 [17]. By the end of cellularization, the basic body plan is determined and the complex cell movements of gastrulation begin. A handful of maternal gene products are spatially patterned in the unfertilized egg in broad gradients along the dorsal/ventral (d/v) and the anterior/posterior (a/p) axes. Zygotic transcription begins at around two hours after fertilization, with the maternal products initiating a hierarchical cascade of transcription factors that drive expression of increasing numbers of genes in more and more intricate patterns. The relatively small number of primary transcriptional regulators that initiate pattern formation (around 40) and the morphological simplicity of the early embryo make the blastoderm a particularly tractable system for modeling animal transcription networks, while capturing the complexities present in all animals.

In this paper, we describe an integrated pipeline of methods for studying gene expression in the *Drosophila melanogaster *blastoderm and release our first set of spatial gene expression patterns digitized from 1,282 embryos. We show that our methods can detect many previously uncharacterized features of morphology and gene expression at a high level of accuracy. An accompanying paper describes further strategies necessary to study temporal changes in gene expression in the presence of dynamic morphology.

## Results and discussion

### A three-dimensional analysis pipeline

**Figure 1:**
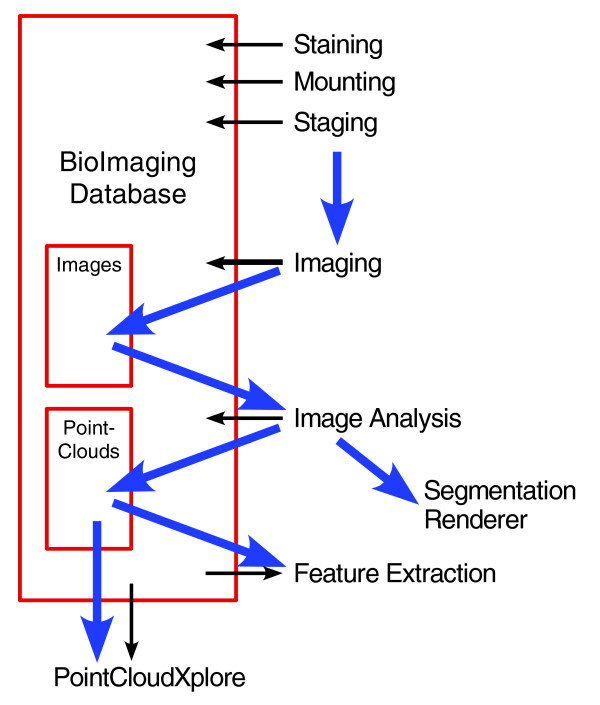
The BDTNP's three-dimensional gene expression analysis pipeline. The major steps of the pipeline are shown. Blue arrows show the path of the major workflow as materials or data files are passed between each step. Black arrows indicate metadata describing experimental details of each step being captured in BID or being retrieved from BID during image analysis, feature extraction, and visualization.

To be able to analyze morphology and gene expression in three dimensions we developed an integrated suite of methods as follows (Figure 1; see Materials and methods). First, embryos were fixed and fluorescently stained to label the mRNA expression patterns of two genes and nuclear DNA, mounted on microscope slides, and visually examined to determine their developmental age. Second, labeled and staged embryos were imaged in whatever orientation they lay on the microscope slide using a two photon laser-scanning microscope to produce three-dimensional images. Third, raw three-dimensional images were converted by image analysis methods into text files, which we call 'PointClouds'. Each PointCloud describes the center of mass coordinates of all nuclei on the embryo surface and the mRNA or protein expression levels of two genes in and around each nucleus. These methods run unattended on large batches of images, processing three to four images per hour, per processor. Fourth, PointClouds were analyzed in three dimensions using a number of automatic and semi-automatic feature extraction methods to determine the orientation of the a/p and d/v axes, record morphological features, measure the locations of gene expression domains, and quantify relative levels of expression. Fifth, a BioImaging database (BID) was employed to track and manage the raw images and PointCloud data files and extensive metadata for each step of the pipeline. Sixth, two visualization tools were used to validate the image analysis methods (Segmentation Volume Renderer) [18], and to analyze the resulting PointClouds (PointCloudXplore) [10,19].

**Figure 2:**
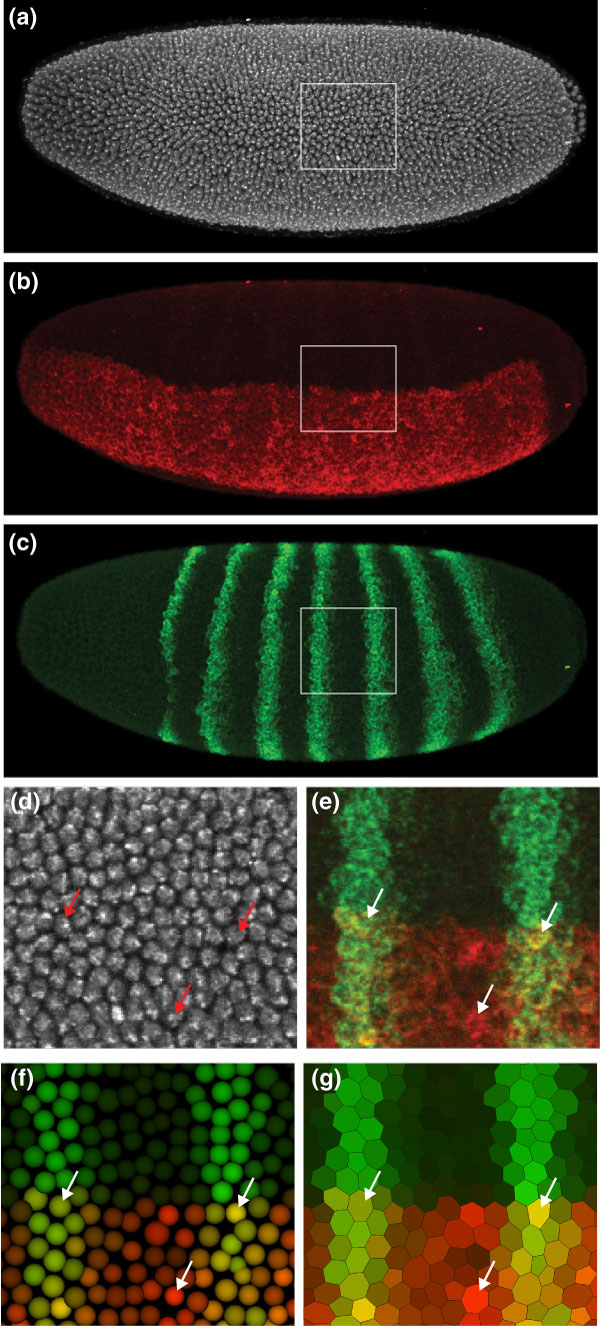
Comparing three-dimensional raw images to PointCloud representations. **(a-c) **Maximum projections of the three channels of a three-dimensional embryo image; **(a) **the nuclear stain (white); **(b) **a *snail *mRNA stain (red); and **(c) **an *eve *mRNA stain (green). Note the small bright speckles visible in all three channels at the same locations. These are outside the cytoplasm and are detected and removed by our image analysis algorithms. The small white rectangles show a region of interest that is displayed in (d-g). **(d,e) **The raw image of the nuclear stain (d) and the mRNA stains for *eve *and *sna *(e). **(f,g) **Two different renderings of the PointCloud derived from this image made using our visualization tool PointCloudXplore: **(f) **uses small spheres whose volumes are proportional to the measured volumes of the corresponding nuclei; **(g) **uses a Voronoi tessellation of the coordinates in the PointCloud. The arrows indicate the locations of the same three cells in each of the panels (d-g).

A critical feature of our strategy is that the large 0.3 to 0.5 Gb raw three-dimensional images for each embryo, such as that shown in Figure 2a-c, are reduced via image analysis to 1 Mb PointCloud files. The resulting PointClouds provide a compact representation of the image data and are readily amenable to computational analysis while maintaining the richness of the blastoderm's morphology and gene expression patterns. Figure 2 provides a qualitative illustration of this, comparing renderings of a part of a three-dimesnional raw image (Figure 2d,e) with two different PointCloudXplore views that represent the same portion of the same embryo (Figure 2f,g). The two mRNA gene expression patterns are well captured on a cell by cell basis in the PointCloud.

### An extensive dataset

To provide an initial dataset for analyses, we used our pipeline to generate 1,282 PointClouds, each derived from a different embryo (Tables 1 and 2). These PointCloud files and their descriptions are publicly available from our searchable BID [10] and cover the expression of 22 genes in embryos from developmental stages 4d (nuclear cleavage cycle 13) and 5. A variety of pair-wise gene combinations are included, but most PointClouds include data for either of the pair rule genes *even-skipped *(*eve*) or *fushi tarazu *(*ftz*), which serve as reference patterns. Data for both wild-type embryos and embryos mutant for three maternal regulators of the early network (*bicoid*, *gastrulation defective*, and *Toll*) are available. We have released more data than used in this and the accompanying paper [20] in the belief that these PointClouds will be generally useful to many researchers and that analysis and modeling of this network will require the combined efforts of a broader community. Data for further genes' mRNA expression, protein expression patterns, mutant embryos, and other *Drosophila *species will be released periodically in the future.

### The challenge of generating three-dimensional PointClouds

Capturing information for the whole embryo in a single PointCloud file posed a number of technical challenges that had to be overcome. We briefly discuss those that are most relevant for understanding of our subsequent analyses. Further details are provided in Materials and methods.

The stage 5 *D. melanogaster *blastoderm is approximately 500 μm along the a/p axis and 150 μm thick at its center. Approximately 6,000 blastoderm nuclei are closely packed around the embryo surface while the interior is filled with opaque yolk granules. The thickness of the embryo and the light scatter caused by the yolk made imaging the complete embryo difficult with standard methods. The close packing of the nuclei required high quality images so that individual nuclei could be resolved. Consequently, fixation, staining, and mounting methods were optimized to maximize stain intensity, preserve embryo morphology, and optically disrupt the yolk granules. Embryos were imaged by laser scanning microscopy using two-photon excitation, which provided superior optical penetration, reduced signal attenuation and higher resolving power along the optical axis compared to single-photon excitation using confocal microscopy [21,22].

The resulting three-dimensional images, however, still suffered from the inherent problems of anisotropic resolution, signal attenuation, and channel cross-talk. To overcome these problems, automated image analysis methods were developed to unmix the fluorescence signals from different channels (Luengo *et al*., manuscript in preparation), correct for signal attenuation and produce an accurate segmentation that defines the position and extent of nuclei detected in the image. (Segmentation is an image analysis term that means to group together pixels that are associated with a particular object in the image.)

**Figure 3:**
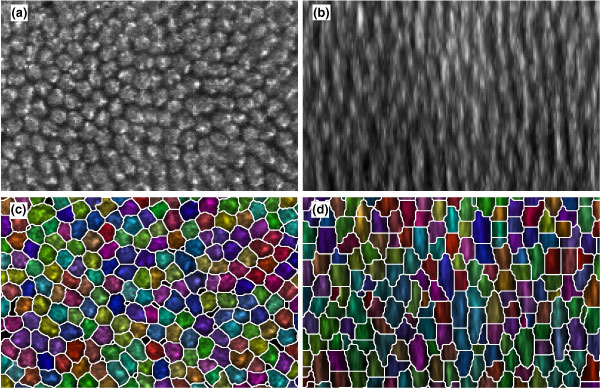
Comparing segmentation results on the top and the side. Using a maximum projection, we show two portions of a three-dimensional image of an embryo fluorescently stained to label nuclei. **(a) **A projection along the optical axis, yielding a *x*-*y *image (the top of the embryo); **(b) **a projection perpendicular to that, yielding a *x*-*z *image (the side of the embryo). The nuclei on the top of the embryo appear well separated and distinct (a). Seen from the side, however, individual nuclei appear elongated along the z-axis due to limited axial resolution, which makes them more difficult to identify (b). The segmentation algorithm provided an accurate segmentation of nuclei **(c) **on the tops of embryo images, but **(d) **on the sides, a model was used to fine-tune the segmentation, resulting in a less accurate result.

An initial segmentation analysis was performed on the image of the DNA stain using a watershed-based method that was constrained using known morphological characteristics of the embryo, such as the fact that nuclei have a polarity perpendicular to the surface of the blastoderm and form a single layer. This strategy identified, on average, 87% of nuclei in an embryo. Most errors occurred in a narrow strip around the embryo where the blastoderm surface is tangential to the microscope's optical axis (that is, on the sides of the image). Visual inspection using our three-dimensional Segmentation Volume Renderer [18] suggests that, outside of these regions, where all nuclei are clearly resolved in the image (Figure 3a), our initial segmentation masks accurately identify the locations of greater than 99% of nuclei (compare Figure 3a and Figure 3c). However, the poorer resolution along the optical axis (compare Figure 3a and Figure 3b) resulted in segmentation errors on the sides of images where two or three nuclei along the optical axis were grouped together. A model based on nuclear size derived from accurate segmentation results in the top and bottom of the image was then used to correct the segmentation errors in these side regions. While the accuracy of this model-based correction was difficult to quantify from the images (compare Figure 3b and Figure 3d), it nevertheless produced segmentation masks that more closely approximated the number and position of nuclei on the sides of images.

To estimate the location of the cytoplasm associated with each nucleus, the nuclear segmentation masks were extended by tessellation laterally until they touched and apically and basally by a fixed distance determined empirically. The nuclear segmentation and the cytoplasmic tessellation masks were then used to record the mRNA expression levels in three regions of each cell: the nucleus, the apical part of the cytoplasm, and the basal part of the cytoplasm. The average fluorescence intensity in one of these three sub-volumes or in the whole cell was selected as the measure of relative gene expression depending on where the mRNA of a particular gene was typically localized within the cell. The recorded mRNA expression levels and the coordinates and volumes of the nuclei and cells were then written in table format as a PointCloud file together with additional metadata describing the embryo's orientation, stage, phenotype, genotype, and staining.

### The landscape of nuclear density patterns

Having established methods to derive PointClouds from image data, we developed a variety of strategies to measure key aspects of morphology and gene expression in three dimensions. Our three-dimensional feature extraction methods not only provided a new quantitative description of the blastoderm, but also yielded a better understanding of the accuracy of our PointCloud representations.

First, we examined the local packing density of nuclei on the surface. Nuclei have long been treated as if they were arranged uniformly around the surface of stage 5 embryos [17,23,24]. Blankenship and Wieschaus [25], however, identified three distinct regions along the a/p axis that had different nuclear densities. Densities were lowest in the anterior of the embryo, higher where the cephalic furrow will later form, and intermediate posterior of this point.

**Figure 4:**
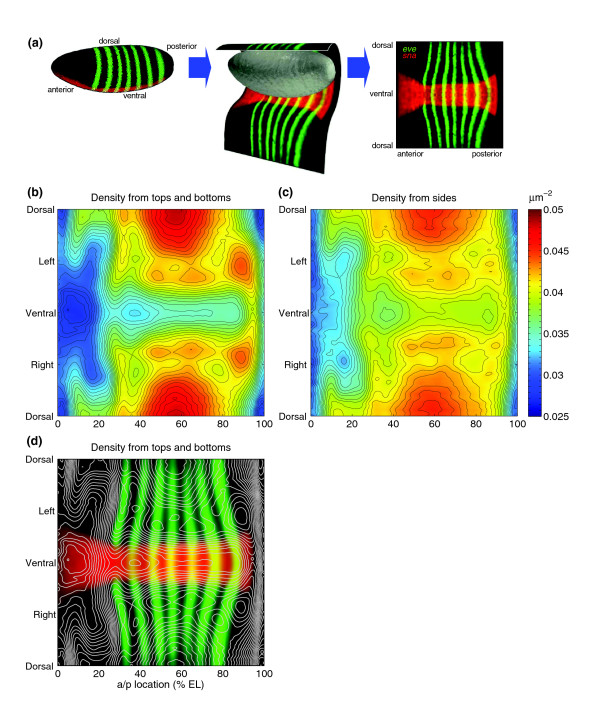
Stage 5 blastoderm embryos show a complex pattern of nuclear densities. **(a) **A schematic representation of how information calculated on the three-dimensional surface constructed from a PointCloud was projected onto a surrounding cylinder and the cylinder was then unrolled to produce a planar map. In these cylindrical projections, anterior is to the left, posterior to the right, the dorsal midline is at the top and bottom, and the ventral midline is in the middle. The distance along the a/p axis is given as a percent egg length (EL). **(b-d) **Average local nuclear density maps were computed from 294 embryos. The maps in (b,d) were computed from the 'top' and 'bottom' portions of each embryo image only, where the segmentation is most accurate. The map (c) was computed from the 'sides' only. The two maps broadly agree, but on the sides of the embryo images the segmentation algorithm has underestimated the number of nuclei dorsally and overestimated the number ventrally. Isodensity curves were plotted over a color map representing local average densities from 0.025 nuclei/μm^2 ^(dark blue) to 0.05 nuclei/μm^2 ^(dark red) (b,c). The average expression patterns of *eve *(green) and *snail *(red) are shown with the isodensity contour (d). The most anterior stripe of *eve *follows a ridge of locally high density, and the boundaries of *snail *expression follow contour lines along about half the length of the embryo.

Based on this observation, we calculated local densities as the number of nuclear centers per μm^2^, measured on the surface of the embryo within the neighborhood of each nucleus. Average values from 294 embryos at late stage 5 were plotted on two-dimensional cylindrical projections to show the densities around the entire blastoderm surface (Figure 4). The embryos were imaged at different, random orientations relative to the microscope objective, each embryo being imaged in one orientation (see Materials and methods). Because the segmentation of nuclei on the tops and bottoms of the images was more accurate, we averaged density measurements from only these higher quality regions (Figure 4b) and, for comparison, measurements taken from only the sides of images (Figure 4c). Since the embryos used for generating the density maps were in many different orientations, using data only from the highest quality regions provided the most accurate assessment of mean densities for all parts of typical embryos.

Our data are in line with the one-dimensional analysis of Blankenship and Wieschaus, but revealed a much more complex, fine-grained pattern of densities that varied continuously around the entire blastoderm surface (Figure 4b). The densities changed by up to two-fold, being highest dorsally and lowest at the anterior and posterior poles, with additional local patches of high or low density also apparent. Some features of the density patterns correlated with the expression of transcription factors that regulate the blastoderm network and with morphological features that form later during gastrulation. For example, the valley of lower density along the ventral midline aligns with the borders of *snail *expression, which also defines the cells that will fold inward to form the ventral mesoderm at gastrulation (Figure 4d). The previously noted ridge of high density that follows the most anterior stripe of *eve *expression (*eve *stripe 1) was also visible (Figure 4d). This region will fold in to form the cephalic furrow just after stage 5 [26]. These density patterns may, therefore, reflect unknown or largely uncharacterized mechanisms that drive later gastrulation movements. Alternatively, they may be merely a non-functional early consequence of gene activities that later cause the larger movements of gastrulation. Whether the nuclear density patterns we observe play a role in morphogenesis or not, they will likely affect the rate at which transcription factors are transported between neighboring nuclei. Thus, they will need to be incorporated into any computational model of this system.

These density measurements also provided an estimate of the accuracy of the segmentation in defining nuclei. The standard deviations of measured density values between PointClouds were between 9% and 18% of the mean. Because the variation between individual PointClouds included all natural variation between embryos and all errors and artifacts introduced at different steps of our pipeline, the standard deviation set an upper limit on the errors our methods introduced. The high reproducibility between independent measurements on the left and right halves of embryos also provided a measure of the accuracy of our analysis (Figure 4b). Finally, to analyze the errors in segmentation on the sides, we computed a density map with data taken from the sides of images (Figure 4c) and compared it to the density map computed with data taken from the tops and bottoms of images. The two maps generated were broadly similar to each other (Figure 4b), and yielded an estimate of the bias in nuclear numbers on the sides compared to the tops and bottoms of images. The maps showed that nuclear numbers were overestimated by up to 11% in the ventral region, and underestimated by up to 7% in the dorsal region when these regions were on the sides of the image.

### Apical/basal nuclear displacement

**Figure 5:**
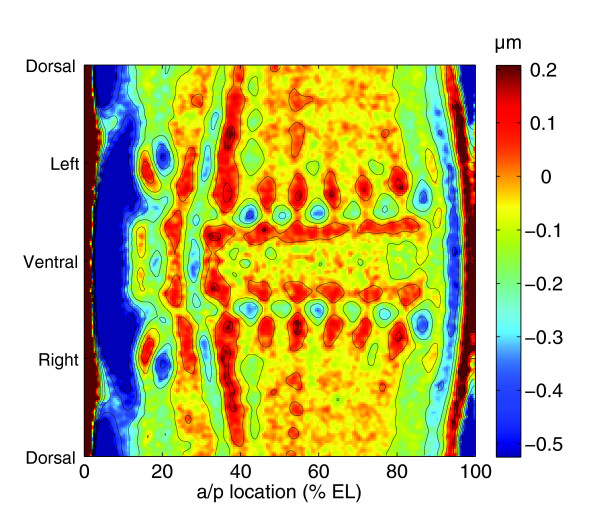
Patterns of nuclear displacement from the PointCloud surface. The location of each nucleus with respect to a smooth PointCloud surface was mapped and averaged over the same cohort of embryos used in Figure 3 and displayed as a cylindrical projection. The map shows that the average apical (positive) or basal (negative) shift of nuclei forms a pattern that appears to correlate with cell fate and the expression patterns of blastoderm transcriptional regulators. Egg length (EL).

While exploring the structure of our PointClouds, we discovered that, during stage 5, the PointCloud surface becomes increasingly rough due to small apical or basal displacements of nuclei. To quantify this, we measured the displacement of each nucleus with respect to a smooth surface fitted through its neighbors (Figure 5). This showed a complex morphological pattern that, like the nuclear density plots, correlated to the expression patterns of transcriptional regulators and later morphological features such as the ventral furrow. The most extreme of these features was an approximate 0.5 μm apical shift above the mean fitted surface, which is equivalent to a single pixel distance in the imaging plane, or about a third of a pixel in the axial direction. Our methods achieved such accuracy because the location of a nucleus in the PointCloud is given by its center of mass, which achieves sub-pixel accuracy. Given the small scale of these movements and the fact that the averages were of a similar order to the standard deviation between individuals (0.7 μm), it is unclear if they have a biological function. However, the ability to measure such small variations demonstrates the sensitivity of our methods, compared to previous analyses that looked by eye for such irregularities prior to gastrulation and failed to detect them, presumably because of their small size [23,27].

### The location of pair rule gene stripes

In addition to morphology, our PointCloud data provided the first opportunity to characterize spatial gene expression patterns in three dimensions. Previous analyses of gene expression in the blastoderm have generally relied on either visual inspection of photomicrographs or quantification of expression stain intensities in narrow one-dimensional strips running along either the a/p or d/v body axes (for example, [6,28]). For our initial three-dimensional analysis, we mapped the locations of the expression stripe borders of three pair rule genes, *eve*, *ftz *and *paired *(*prd*), that are a key part of the cascade that determine cell fates along the a/p axis. First, we divided the embryo surface into 16 strips running along the a/p axis that were evenly spaced around the embryo circumference. For each strip, inflection points were then used to estimate the location of stripe borders along the a/p axis. The inflection point of a slope is defined as its steepest point (that is, a local maximum in the derivative).

**Figure 6:**
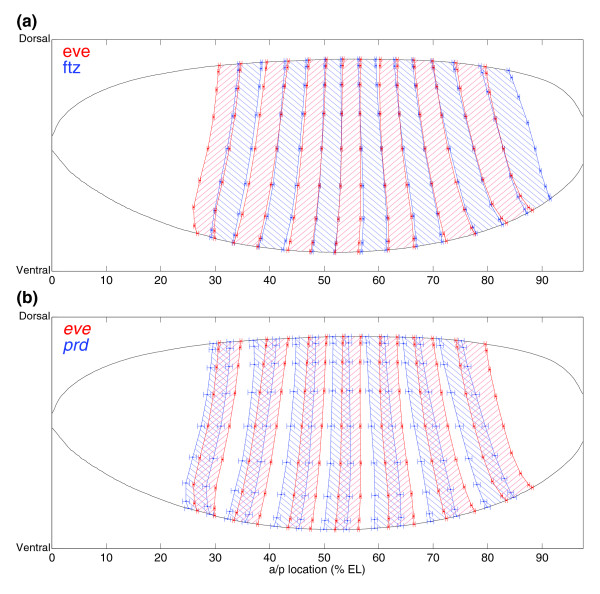
Locations of stripes of the pair rule genes *ftz*, *eve *and *prd*. The locations of stripe borders along the a/p axis were computed at 16 locations around each embryo; the measurements for all embryos were averaged. The results are displayed as orthographic projections in which the anterior of the embryo is to the left and the dorsal midline to the top. Pair-wise comparisons of the expression of **(a) ***eve *and *ftz *and **(b) ***eve *and *prd *are shown. The error bars give the 95% confidence intervals for the means. The relationship between *eve *and *ftz *stripes was constant, but *prd *stripes shifted their registration relative to *eve*'s along both the a/p and d/v axes. The data for *eve *expression were derived from *n *= 215 embryos at stage 5:50-100%, *ftz *from *n *= 155, and *prd *from *n *= 17. Egg length (EL).

Figure 6 plots the stripe border locations in two-dimensional orthographic projections. The data show that at approximately 57% egg-length the pair rule stripes maintained a relatively constant a/p position around the embryo circumference as measured in each of the 16 strips. This was not the case, however, for the stripes more anterior and posterior of this point. Between the dorsal and ventral midlines, stripes were displaced by up to 9.3% egg length (for example, *eve *stripe 7), or approximately 7 cell diameters. Furthermore, our data show that the stripes are curved, not straight.

The fact that a/p positions of pair rule stripes vary along the d/v axis has long been apparent from visual inspection of low resolution two-dimensional data (for example, [29]). The nomenclature commonly used to describe the blastoderm system, however, does not easily accommodate this displacement. Pair rule genes are often said to specify position only along the a/p axis. Yet, using the traditional definition that the d/v and a/p axes are straight and perpendicular to each other, the relative locations of pair rule stripes clearly change along both axes and thus have the potential to specify information along the d/v axis also. For example, a line orthogonal to the a/p axis at 80% egg length passes from *ftz *stripe 7 at the dorsal midline, across *eve *stripe 7, to the center of *ftz *stripe 6 at the ventral midline (Figure 6). For pair rule genes to be said to only specify the a/p position, the principal body axes would have to be redefined in such a way that they curve to follow stripe expression. While we do not necessarily advocate such a coordinate system, as we show later, it is at times convenient to derive measures by following gene expression features around the circumference of the embryo, rather than along a straight body axes.

We also found that pair rule genes do not always maintain the same register along the a/p axis. When *eve *and *ftz *stripes were compared, they had largely non-overlapping complementary patterns that do maintain the same registration relative to each other, both along the a/p axis and around the circumference of the embryo, consistent with previous reports [30] (Figure 6a). In contrast, the registration between *eve *and *prd *stripes changed. For example, *prd *stripe 1 has a much larger overlap with *eve *stripe 1 than *prd *stripe 7 has with *eve *stripe 7. In models of pair rule regulation, gene expression patterns are typically said to maintain spatial registration (for example, [31-35]). Clearly this is not always the case, implying that the rules that govern regulatory networks are more subtle and complex than current models suggest.

As was the case with measurements of morphology, these stripe feature extraction measurements also provided an indication of the accuracy of our methods. The 95% confidence limits along the a/p axis (Figure 6) are small compared to the stripe displacements noted, indicating that the changes observed are significant in our assays.

### Measuring relative intensities of gene expression stripes

One of the strongest motivations for developing our gene expression analysis pipeline was the desire to obtain quantitative descriptions of gene expression levels. It is well known that the expression of transcription factors changes quantitatively from cell to cell and that this results in quantitative responses in the rate of transcription of their targets (for examples in the *Drosophila *blastoderm, see [6,36,37]). Our methods cannot precisely capture absolute levels of gene expression, largely due to variations in labeling efficiency between embryos and microscope performance. At a minimum, however, we ought to capture relative levels of expression, which should be adequate for determining regulatory relationships between transcription factors and their targets.

We addressed three questions to help establish how well our methods provide a quantification of relative expression. First, did our attenuation correction correctly overcome the problem of signal attenuation through the depth of the embryo to provide reliable quantification in three dimensions? Second, did our enzyme-based mRNA labeling methods give quantitatively similar results to antibody-based labeling of protein, which is generally viewed as giving fluorescence intensities proportional to expression levels? Third, was our quantification of expression patterns sufficiently consistent between embryos that relative expression patterns for each gene could be determined?

**Figure 7:**
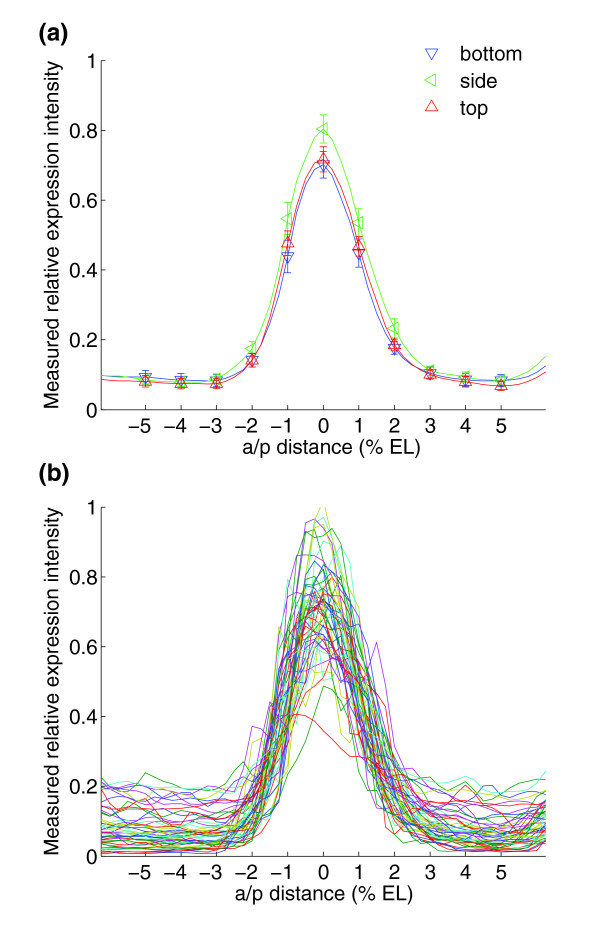
Expression intensity profiles taken from embryos imaged in different orientations. **(a) **The average intensity profile measured on the image bottom (blue), side (green) or top (red) with respect to the orientation of the embryo in the microscope. Intensities for *eve *stripe 1 were measured within two strips 1/16th of the width of the embryo circumference located on the left and right lateral midlines, after normalizing the expression values by setting the 1st percentile of the values in the whole embryo to 0 and the 99th percentile to 1. The plot shows the average intensity along the a/p axis for these strips. The difference in height between the three graphs gives an indication of the orientation-specific error. The measured intensity differs by less than 10% when the embryo surface is perpendicular or parallel to the optical axis. **(b) **An indication of the variation between individual PointClouds; the 52 profiles used to obtain the *top *average profile in (a).

The accuracy of our attenuation correction was simple to test because the corrected gene expression levels we derived must be independent of the orientation of the embryo when it was imaged. Therefore, we compared expression intensities at the same location on the same stripe for multiple embryos imaged in different orientations. We compared the average levels of expression at the left and right lateral midlines of a single *eve *expression stripe. Expression was averaged from a group of 52 embryos where the lateral portions of the embryo were at the top and bottom of the embryo relative to the microscope objective, and 31 embryos where these regions were on the side. The average expression level was plotted along the a/p axis, giving a profile of the rising and falling level of expression across the width of a stripe. Figure 7a shows that mean expression profiles for the *top *and *bottom *groups were indistinguishable, indicating that the attenuation correction was accurate. But the *side *group had a peak of expression at the center of the stripe about 10% higher, indicating a modest error in quantifying expression at the sides of the image. We suspect that this error was caused by blurring along the optical axis. This distributes expression fluorescence signal from one cell to its neighbors on the sides of the image, and from one cell to the background on the top and bottom of the image. Since this error is small and known, more accurate estimations of expression could be achieved by averaging data from embryos in a variety of orientations or, if desired, by weighting against data derived from the sides of three-dimensional images or building an explicit model to correct for this error.

**Figure 8:**
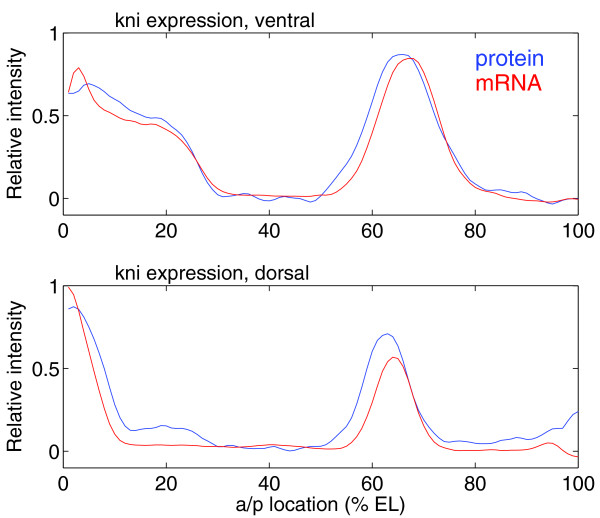
Methods for quantifying relative protein and mRNA levels give similar results. Average expression of *kni *mRNA at the beginning of stage 5 (7 embryos) is compared to *kni *protein expression at mid-stage 5 (17 embryos). The two graphs show the expression along the a/p axis (x-axis) at the ventral (top graph) and dorsal (bottom graph) midlines. The levels of fluorescence for mRNA labeling and protein labeling have remarkably similar shapes. Egg length (EL).

The method we used to fluorescently label mRNA expression patterns included a signal amplification step with horseradish peroxidase enzyme that, to our knowledge, has not been shown to yield fluorescent product in proportion to the amount of mRNA. In contrast, protein stains with fluorophore-conjugated antibodies are generally considered to be a proportional measure of protein expression levels, and a recent analysis by Thomas Gregor *et al*. has confirmed this assumption (T Gregor, E Wieschaus, A McGregor, W Bialek, and D Tank, personal communication). As an indirect test of whether our mRNA detection method provides a linear measure of RNA concentration, we compared the relative levels of mRNA and protein for one gene, *knirps *(*kni*). Because protein expression patterns lag mRNA expression patterns in time, we compared expression of mRNA in early stage 5 embryos to protein expression at mid stage 5. As Figure 8 shows, the relative levels of expression of *kni *protein and mRNA closely match. Thus, our mRNA detection methods and antibody-based protein detection methods appear to be similarly quantitative.

To examine the consistency of our quantification methods across embryos, we examined the variation in expression levels between measurements from individual PointClouds (Figure 7b). Multiple factors contributed to this variation, including natural variation between individual embryos and a range of inaccuracies that could have been introduced by our pipeline, such as differences in scaling, background staining, imaging noise, and segmentation errors. Given this, the similarity of the data was reassuring and suggested that our data were a useful guide to relative gene expression.

### Pair rule expression within stripes varies around the d/v axis and is different for adjacent stripes

**Figure 9:**
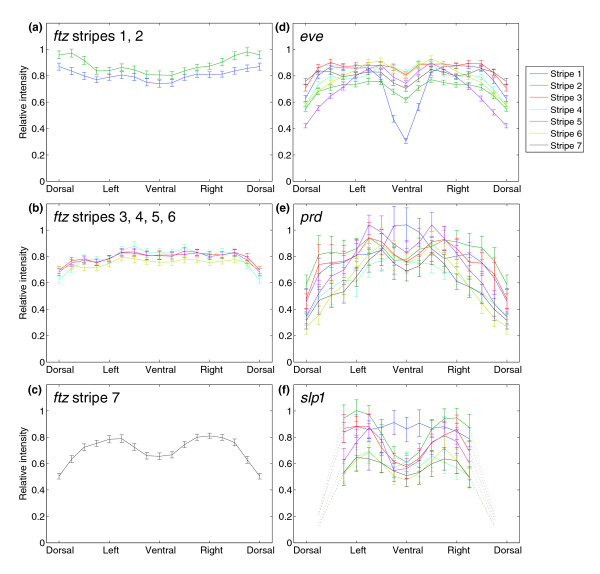
The relative levels of pair rule stripe expression vary between and along stripes. Plotted are averaged expression intensities of gene stripes for **(a-c) ***ftz*, **(d) ***eve*, **(e) ***prd *and **(f) ***slp1*. The various stripes of each gene show marked differences in expression profiles and each gene has a unique mode of variation in the direction of the d/v axis. The error bars give the 95% confidence intervals for the means. The data for *eve *expression were derived from *n *= 215 embryos at stage 5:50-100%, *ftz *from *n *= 155, *prd *from *n *= 17, and *slp1 *from *n *= 23.

To further explore the consistency of our quantification, we compared expression levels for each stripe for several pair rule genes. We first measured the local maximum intensity in different regions around the circumference of the embryo within each stripe. In other words, expression was compared along the stripe in the direction of the d/v axis, but not along the straight line of the d/v axis so as to avoid the complication caused by the three-dimensional shape of the stripes. As Figure 9 indicates, our methods showed clear quantitative differences in expression both between stripes and within individual stripes in the direction of (but not along) the d/v axis. The fact that these differences are less than the 95% confidence limits for the mean intensity shows that our methods are sufficiently consistent to detect these variations.

In the case of *ftz*, the expression profiles of stripes 1 and 2 were similar to one another; those of stripes 3 to 6 were also similar, but the profiles of both of these groups of stripes differed from one another and from stripe 7 (compare Figure 9a-c). Stripes for *eve*, *prd *and *sloppy paired *(*slp1*) also showed different relative levels of expression, and there was no apparent relationship between equivalent stripes for each of these genes. The magnitudes of many of these differences in expression were up to and, in some cases, greater than two-fold. There are many precedents for changes in transcription factor concentrations of this magnitude affecting the control of downstream target genes, such as the effect of *eve *concentration on *ftz *[37] or the number of bcd copies on its target genes [36,38]. Thus, it is quite possible that these changes in pair rule expression will have a functional impact on the network.

**Figure 10:**
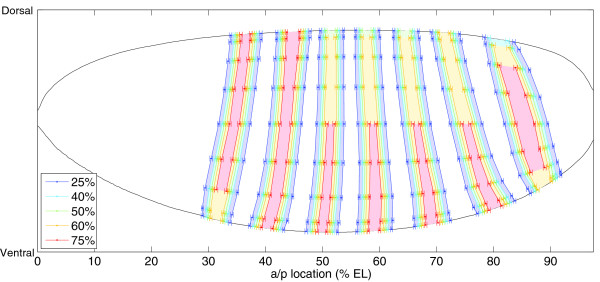
The boundaries of relative levels of ftz expression. Plotted are the averaged locations of various threshold levels of *ftz *expression derived from 155 embryos, computed and displayed similarly as in Figure 6. For example, those cells expressing *ftz *above 75% of the maximum level of expression are shown in red. Note the shape of the stripes above the 50% threshold is similar to that given by the inflection points (Figure 6), but not equal. For example, the dorsal-most point of stripe 7 is less than 50% of the maximum expression level for more than half the embryos (that is, the stripe at that point is not shown in this graph). Egg length (EL).

Figure 10 provides another view of this d/v modulation, showing that the spatial pattern proscribed by expression of *ftz *above a given threshold does not specify a constant width segment of cells. The highest levels of *ftz *expression do not even specify the full stripe around the circumference of the embryo; see, for example, the group of cells expressing *ftz *above 75% of the maximum level.

### Implications for the specification of positional information by pair rule genes and the interplay of the a/p and d/v regulatory systems

The principal biological function of each pair rule gene is presumed to be to specify repeated locations within the embryo, each stripe specifying (at least to a first order approximation) the same information. Although qualitative differences in expression levels around the embryo circumference for individual stripes of pair rule genes have been noted in a few cases previously (for example, [39,40]), in general, little consideration has been given to changes in expression either between equivalent positions on different stripes or between different locations within stripes in the direction of the d/v axis. The variation in stripe position and expression levels suggests that genes whose principal function is to specify expression along the a/p axis have the potential to also convey some modest patterning information along the d/v axis.

Conversely, the fact that pair rule gene expression changes quantitatively in the direction of the d/v axis also implies that, directly or indirectly, d/v axis regulators, such as *twist*, *snail *and *dorsal*, are responsible for generating these changes. As we show in the accompanying paper [20], this is the case. The regulatory systems controlling the two principal body axes appear to mutually interact early during zygotic transcription.

## Conclusion

The *Drosophila *blastoderm embryo is one of the most intensely studied systems in developmental biology, both in the areas of transcriptional regulation and morphological development. The fact that our three-dimensional methods have quickly uncovered new features of this system suggests there is still much to learn about many developmental processes. The detailed complexity of morphology and gene expression revealed by these methods, much of which cannot be readily judged by eye, suggest that quantitative three-dimensional measurements and computational analyses will be essential if we are to truly describe and understand animal regulatory networks.

The methods we have presented here and in the accompanying paper are by no means sufficient, however. Further work will be required to establish how well our data capture levels of gene expression. The dataset we have released provides information for individual embryos, each showing the expression of only a pair of genes. To examine regulatory relationships between transcription factors and their targets, it will be important to compare the expression of many genes within a common framework [41,42]. To this end, we have developed methods for aligning information from multiple PointClouds to allow such cell-by-cell comparisons of the expression of hundreds of genes and are using these to explore the relationships between regulator and target gene expression patterns (CC Fowlkes and J Malik, unpublished data). In addition, our methods will require further development before they can be applied to the analysis of gene expression in later stages of *Drosophila *development and to other animal systems. The broader application of quantitative three-dimensional analyses will likely require the efforts of a large multidisciplinary community of researchers.

## Materials and methods

### Fly stocks and nucleic acid probes

Wild-type embryos were cultured in cages for many years, starting with a nominally CantonS strain.

Full length *eve*, *ftz*, *gt*, *hb*, *kni*, *Kr*, *prd *and *slp1 *cDNAs were inserted in Gateway pDEST-vectors (M Stapleton, B Grondona, unpublished data). A 1.7 kb Sna cDNA fragment in pBSK(+) was a gift from E Bier (UC Santa Cruz, CA, USA). To create linear DNA templates, pDEST full length cDNAs were amplified using extended vector primers such that the T3 primer sequence was 3' of the cDNA and the T7 primer lay 5' (T7: 5'-GTA ATA CGA CTC ACT ATA GGG ACA TCA CCT CGA ATC AAC A; T3: 5'-AAT TAA CCC TCA CTA AAG GGC GGG CTT TGT TAG CAG C). The pBSK+ cDNA was PCR-amplified using M13 ± primers. Antisense biotin (BIO), digoxigenin (DIG) or dinitrophenyl (DNP)-labeled RNA probes were prepared by *in vitro *transcription from PCR generated DNA templates for each gene using T3 RNA polymerase. To increase signal, the probes were not hydrolyzed [43].

### Fluorescent triple-staining

Wild-type embryos were collected for 1 h and matured for 3 h at 25°C, then dechorionated with 50% household bleach for 3 minutes and fixed for 20 minutes with 1:4 (v/v) solution of 10% formaldehyde (Polysciences, Warrington, PA, USA) and heptane (Sigma, St. Louis, MO, USA). Fixed embryos were devitellinized by shaking vigorously in 1:1 methanol/heptane, after which they were washed three times with methanol and once with 100% ethanol, and stored in ethanol at -20°C.

Embryos were rehydrated in phosphate buffered saline pH 7.2, 0.05% Tween20, 0.2% TritonX-100 (PBT+Tx), post-fixed for 20 minutes in 5% formaldehyde/PBT+Tx, and, after several washes in hybridization buffer (50% formamide, 5 × SSC pH 5.2 to 5.4, 0.2% TritonX-100, 50 μg/ml heparin) at 55 to 59°C, prehybridized for 1 to 5 h in hybridization buffer. There was no proteinase K treatment. To improve the staining quality, the prehybridized eggs were stored in -20°C hybridization buffer for at least 16 h.

For each *in situ *hybridization, 50 to 100 μl of embryos were incubated in 300 μl of hybridization buffer with an RNA probe for one gene labeled with DIG and an RNA probe to a second gene labeled with either DNP or BIO. After 12 to 48 h co-hybridization at 55 to 59°C and several high-stringency and low stringency washes, the two probes were detected sequentially. The DIG-labeled probe was detected using 1:500 horseradish peroxidase conjugated anti-DIG-antibody (anti-DIG-POD; Roche, Basil, Switzerland) and either a Cy3 or coumarin-tyramide reagent (Perkin-Elmer TSA-kit, Wellesley, MA, USA). Before the second probe was detected, the anti-DIG-POD antibody was first removed with several 15 minute washes with 50% formamide, 5 × SSC, 0.2% TritonX-100 at 55°C, followed by inactivation of the remnants with 5% formaldehyde/PBT+Tx. Then the second probe was detected using 1:100 anti-DNP-HRP (Perkin-Elmer) and either the complementary coumarin or Cy3-TSA-tyramide reaction. To allow detection of nuclei with a nucleic acid binding stain, all RNA in the embryo was first removed by digestion with 0.18 μg/ml RNAseA in 500 μl overnight at 37°C, and then the DNA was stained overnight by incubation in 500 to 1,000 μl of a 1:5,000 dilution of Sytox Green dye (Molecular Probes, Carlsbad, CA, USA).

The kni protein expression was detected with guinea pig-anti-kni (a gift from J Reinitz, Stony Brook University, Stony Brook, NY, USA) and Alexa488-anti-guinea pig (Molecular Probes) in embryos hybridized against *ftz *DIG-mRNA that was detected with coumarin tyramides. For these embryos only, the nuclei were detected using mouse-anti-histoneH1 and Alexa555-anti-mouse.

The stained embryos were dehydrated with an ethanol-series and mounted in xylene-based DePex (Electron Microscopy Sciences, Hatfield, PA, USA). A #1 coverslip was placed on a bridge formed by two #1 coverslips to prevent embryo flattening. This mountant has the advantages of creating permanent slides that protect the fluorophore from oxygen, which makes the samples highly resistant to photobleaching. To estimate the refractive index of the mountant (which determines the scaling of the z-axis), we used the assumption that embryo morphology was independent of the orientation of the embryo when it was imaged. A d/v cross-section of multiple embryos was taken at 50% egg length. Within these cross-sections, the ratio of the d/v length to the left/right length was plotted against orientation angle (data not shown). The refractive index was then computed so that this ratio was independent of the orientation. The average refractive index calculated using this method was 1.62 ± 0.06.

### Temporal staging

Each of the imaged embryos was individually staged from a phase contrast view and the stages were recorded into BID. Embryos of stage 5 [17] were subdivided into cohorts based on the degree to which membranes had invaginated during cellularization. For example, an embryo in which the cellular membranes had invaginated 50% of the distance across the cortical cytoplasm would be staged as stage 5:50%. Because the rate of cellular invagination varies along the d/v axis, being most rapid ventrally, the percentage of membrane invagination was visually estimated where possible at the ventral surface of the embryo. If the embryo was lying in an orientation where the ventral surface was not visible in cross-section, however, we estimated the degree of membrane invagination at that side of the embryo where invagination was most advanced. Later, the stage of these embryos was corrected based on our observation that membrane invagination is about 70% laterally when it is at 100% ventrally, yet at 40% invagination it is approximately even all around the embryo. The degree to which membranes had invaginated ventrally was estimated using a linear mapping for cases where membranes had invaginated laterally at least 50% using the function 50 + (5/2)(*v *- 50) (where *v *is the lateral invagination percentage). The d/v orientation of all embryos was determined from their respective PointClouds based on gene expression features (see below). For the analyses presented in this paper, we used embryos in the range stage 5:50-100% invagination, which is a time window of 10 to 15 minutes [44].

### Imaging

Three-dimensional images of the whole embryos were obtained on a Zeiss LSM 510 META/NLO laser scanning microscope (Carl Zeiss MicroImaging, Inc., Thornwood, NY, USA) with a plan-apochromat 20×, 0.75 numerical aperture objective. This objective allowed imaging of entire embryos in a single field-of-view while providing sufficient resolution and sensitivity for the subsequent analyses. The fluorophores were excited simultaneously by dual 750 nm photons supplied by a Chameleon laser (Coherent, Inc., Santa Clara, CA, USA). The resulting emission spectrum was split by dichroic mirrors and collected by three independent photomultiplier tubes (PMTs). The signals were digitized into 12 bits and recorded as three-channel images, each of a size up to 1,024 by 1,024 by 150 pixels, which varied depending on the embryo size. Each pixel had a transverse dimension of 0.45 μm and an axial dimension of approximately 1.6 μm, which varied slightly with the refractive index of the mounting medium. The gain and offset of the PMTs were set so that all the pixels of interest fell within the 12 bit dynamic range.

### Segmentation

The position and extent of the nuclei on the surface of the embryo were defined by a model-based three-dimensional segmentation analysis. Here we discuss some of the main aspects of the algorithm. All image processing and analysis algorithms were implemented in MATLAB (The MathWorks Inc, Natick, MA, USA) with the DIPimage toolbox [45,46].

**Figure 11:**
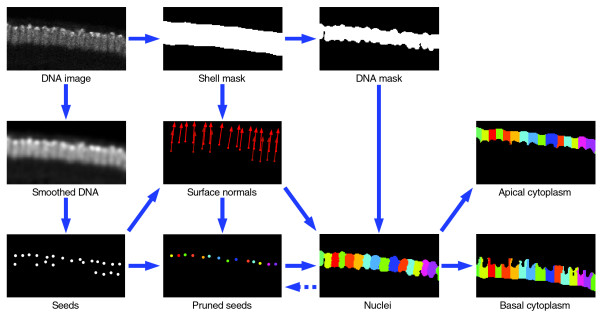
Overview of the segmentation algorithm. The main steps of the algorithm are illustrated here on a small portion of a slice through the middle of an embryo. Note that the actual images are three-dimensional and comprise a whole embryo. The DNA image is the input Sytox channel. A shell mask defines the region that contains all the information of interest for the segmentation algorithm: the blastoderm nuclei with a small part of the cytoplasm. The DNA mask distinguishes the nuclei from the background (cytoplasm, yolk, and so on). The seeds image contains the local maxima of the smoothed DNA, a Gaussian filtered version of DNA image. Surface normals are computed for each seed from the shell, and used to prune the seeds. The image nuclei is the nuclear segmentation mask, dividing the DNA mask into individual nuclei. The dotted arrow going back to the pruned seeds represents the addition of seeds according to the results obtained in nuclei. The apical cytoplasm and basal cytoplasm mark the cytoplasmic regions for each nucleus estimated using a tessellation.

**Figure 12:**
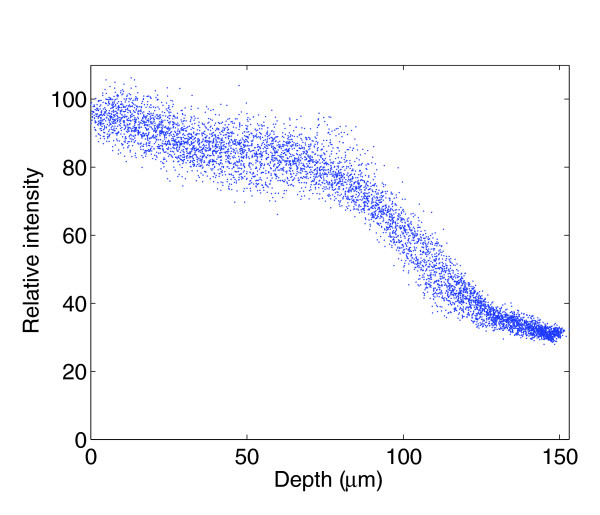
Sytox attenuation with depth. Relative intensity of the Sytox stain within each nucleus, plotted against the depth of the nucleus along the optical axis. Sytox levels were normalized by scaling the 99th percentile of intensity to 100.

The segmentation routines used as input the image of the Sytox DNA stain channel, labeled 'DNA image' in Figure 11. To restrict the analysis to the nuclei on the embryo surface, a three-dimensional binary mask, the 'shell mask' (Figure 11), was defined around the embryo surface by taking an adaptive threshold of the 'DNA image' that varied on a per-slice basis to account for signal attenuation (Figure 12). This shell mask was used to direct spectral unmixing of the Cy3, Sytox and Coumarin channels. It also allowed the initial attenuation correction of the Sytox channel required for the segmentation. This was accomplished using a local contrast stretch within the shell mask. A global threshold was then applied to the unmixed, attenuation-corrected Sytox channel, which was then masked by the shell image. The resulting 'DNA mask' (Figure 11) identified the regions in the image that belong to the blastoderm nuclei.

To locate individual nuclei, the DNA image was convolved with a narrow Gaussian to reduce noise. Local maxima in the resulting image, termed 'seeds' (Figure 11), were then used to determine nuclear position. Multiple seeds were often observed in a single nucleus along its apical-basal axis on the sides of images, due to anisotropic resolution and nuclear geometry. Multiple seeds were also occasionally detected on the bottom of the embryo, where the signal to noise ratio was low due to signal attenuation. To eliminate multiple seeds, the embryo 'surface normal' for each seed was computed by applying the structure tensor [47,48] to the three-dimensional skeleton [49-51] of the shell mask(Figure 11). Neighboring seeds that lay along this normal were assumed to belong to the same nucleus and simply removed, leaving only a set of 'pruned seeds' (Figure 11).

Once a single seed was determined per nucleus, the pruned seeds were grown to fill the nuclei, using a region-growing algorithm that combined a watershed algorithm [51,52] and a gray-weighted distance transform [51,53,54] of the DNA image (Figure 11). The combination of these two algorithms created nuclear boundaries that matched actual boundaries when visible, yet divided distances between seeds equally when boundaries where not distinguishable.

In some cases nuclei, predominantly on the sides of images, did not posses a seed and were joined to one of its neighbors. These regions were detected by comparing their sizes to average sizes taken from the top and bottom of the image where segmentation was most accurate (Figure 3). The original seeds for these regions were then replaced by an appropriate number of seeds using a cluster analysis algorithm [55] that placed seeds on the brightest possible locations that created regions of similar total intensity. The region growing algorithm described above was executed again on this refined set of seeds. Finally, regions that were still too large were just split into an appropriate number of equal volumes without regard for the pixel intensities. Our Segmentation Volume Renderer [18] was used extensively during the development of the segmentation algorithm.

Finally, the segmentation algorithm includes additional features that make it more robust to images with specific artifacts that would have otherwise resulted in failure to generate a PointCloud. One example is the occasional presence of impurities on the embryo surface that caused a bright artifactual fluorescence signal across all channels. These regions were detected in the image and ignored during subsequent analysis. A second example is the occasional presence of a yolk nucleus proximal to the blastoderm nuclei. Such a yolk nucleus results in a shell mask with a local basal bulge. This condition was simply detected and removed. For full details on the segmentation algorithm refer to the source code, available online [10].

### Measuring expression levels associated with each nucleus

To capture the labeled mRNA expression levels, we first had to estimate the cellular extent surrounding each nucleus. This was achieved by growing the nuclear segmentation mask, in the apical and basal directions, into the cytoplasm by tessellation. The distances grown were established by examining cytoplasmic auto-fluorescence in several sample images. This was then used in combination with the nuclear mask to divide each cell into three regions: apical, nuclear and basal (Figure 11). The expression level was estimated in each of these regions and in the whole cell by taking the average values within them for both the Cy3 and Coumarin channels. These expression values, together with the average value of the Sytox channel within each nucleus, the center of mass of the nuclei, the volumes of the various cellular regions, and the neighborhood relationshps between cells were written to a PointCloud file.

For subsequent analysis, expression values from the PointClouds were corrected for attenuation by dividing these values with the average Sytox intensity within the corresponding nucleus. This approach assumes that the average Sytox intensity is constant from nucleus to nucleus, and it is representative of the attenuation of the other channels.

### Cylindrical and orthographic projection of the blastoderm

We use two methods to display data on the embryo surface: the cylindrical projection and the orthographic projection. The cylindrical projection provides an 'unrolled' view of the full surface, which we use to display data mapped onto the blastoderm surface. The orthographic projection shows only half the surface, but produces less distortion and, therefore, is useful to show the location of borders of the a/p patterning system. The center of mass of the embryo was computed from the three-dimensional nuclear coordinates in the PointCloud as the mean coordinate of all points. The principal a/p axis of the embryo was estimated as the eigenvector associated with the smallest eigenvalue of the inertia tensor [47]. The location of the dorsal-most point was determined manually for each PointCloud from the *ftz *or *eve *expression pattern. The embryo was then translated so that the center of mass was at the origin, and rotated so that the estimated a/p axis lay on the x-axis and the d/v axis lay on the z-axis, anterior to the left (negative x), dorsal up (positive z). The cylindrical projection then used the *x*-coordinate on the horizontal and *ϕ *on the vertical, where *y *= *r *sin(*ϕ*) and *z *= *r *cos(*ϕ*). This resulted in a rectangular plot with the embryo's anterior to the left, the dorsal midline split to the top and bottom, and the ventral midline in the middle. Orthographic projections simply used the *x*-coordinate on the horizontal and the *z*-coordinate on the vertical, discarding *y*. As a further aid in managing the complexity of this three-dimensional dataset, we developed a flexible visual analysis tool, PointCloudXplore [19], which can be used to interactively visualize and analyze the embryo PointClouds in three dimensions.

### Computing packing density of nuclei

Nuclear packing densities were calculated as the number of nuclei per unit surface area. The surface of the embryo was first identified from the PointCloud using the Eigencrust algorithm [56]. Briefly, a region was defined by sweeping a 15 μm arc on the embryo surface about each nucleus. The density was then estimated as the number of nuclei inside this region divided by its area. Average density maps were computed by resampling the per-nucleus density estimates for a given embryo onto a regular grid in cylindrical coordinates, and averaging these resampled projections over the embryos in a temporal cohort. Only the top and bottom parts of the z-stacks were used for density analyses, except for method evaluation comparison in Figure 4c, where the laterals of the z-stacks were used.

### Computing apical/basal shift of nuclei

Apical/basal shift was measured by fitting, using least squares, a quadratic surface to the 200 nearest neighbors of a nucleus, and determining the distance of the nucleus to this surface. Average shift maps were computed using resampled cylindrical projections, in the same manner as the average density maps. To eliminate the possibility that bleed-through from mRNA stain channels might influence the segmentation and localization of nuclei in the Sytox channel, we also examined average shift maps produced from subsets of embryos excluding those embryos stained for particular genes (data not shown). All of these maps showed qualitatively similar patterns of nuclear displacement.

### Measuring expression boundary location

To determine an initial estimate of the boundary location, two algorithms were created to find the approximate location of the pair rule and gap gene stripe boundaries from PointCloud data. The first algorithm was fully automatic, once the number of stripes was specified. It used a local threshold to detect regions that contain the highest expression values. The edges of these regions provided approximate locations for stripe boundaries. A second semi-automatic algorithm was developed for immature patterns (such as the early *ftz *pattern), and those that did not segment properly because of imaging artifacts. In these cases, a user clicked on a nucleus close to the stripe border of interest. The shortest geodesic path [57] that circumnavigated the embryo through this point was determined. This was done using a gray-weighted distance transform [51,53,54] of the gradient of the stripe expression pattern, taken along the a/p direction, and resulted in a path that followed the stripe edge. When this failed, the stripe boundary was determined manually by placing eight points on each edge.

To compute the location of the stripe boundaries, the embryo was first divided into 16 equal strips running along the a/p axis. Nuclei that fell within each strip were projected onto the a/p axis and their expression values were sampled at 400 regular intervals, using normalized convolution [58] with a Gaussian of *σ *= 1 interval (this yields 16 one-dimensional graphs). Accurate boundaries of expression stripes were then determined by finding the center of mass of peaks in the gradient of expression along the strip. The center of mass was used because it is more robust against noise than the expression gradient maximum, which marks the expression inflection point, a feature commonly used to mark edges.

For Figure 10 where the boundaries were computed using a threshold, we thresholded the one-dimensional projections of the 16 strips as defined above, then determined the location of the boundary closest to the expected boundary location, as given by the inflection points. Due to variation between individuals, some embryos did not posses all points used in this graph. The measurements at each point were averaged for all embryos that possessed a threshold at that point. Where more than 50% of embryos lacked a point, that point was not shown.

### Measuring stripe intensity

The intensity of pair rule gene stripes was determined using the 95th percentile of the expression level values (as a more robust substitute for the maximum), within a region determined by the 1/16th strip and the stripe borders as determined above.

### Data management and storage

**Figure 13:**
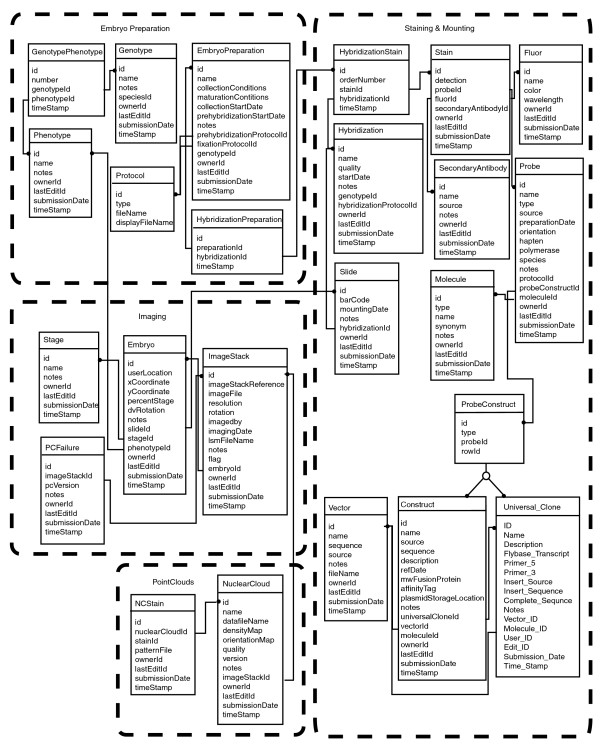
BID schema. Each table corresponds to a step in the experimental process. The tables have been grouped into four blocks corresponding to a coarser subdivision of the pipeline.

A BID was built to manage and store of all the data and metadata produced by this project [10]. BID tracks the entire experimental process from the embryo preparation (genotype, phenotype, collection conditions, maturation conditions, and so on) and hybridization (nucleic acid probes, secondary antibodies, fluorophores, and so on, including detailed information such as the vector DNA sequence), all the way to the PointCloud data files (with associated metadata such as a quality score, thumbnails and links to the raw image data). For each step in the experimental process, a corresponding table or set of tables describes the fine-grained details of that process (Figure 13).

Sophisticated search functions and overviews of the experiments are provided to aid project management. For example, it is possible to quickly find the slide and embryo location for a given PointCloud, should it need to be re-imaged or re-staged. This is accomplished by identifying each slide with a unique bar code and each embryo that was imaged by its coordinates on the slide. For a full schema see Figure 13.

The raw three-dimensional images are stored in a dedicated repository, and indexed with BID. Because of their large size (approximately 400 Mb each), the raw images require a different backup solution as well as a high-speed network between the storage and the computers used for processing them. The independent repository makes this possible.

## Figures and Tables

**Table 1:** Number of genes' mRNA expression patterns from individual PointClouds in Release 1 for the series of developmental stage cohorts used in [20]

Wild type	Stage cohort							Total
		
	4d	5:0-3%	5:4-8%	5:9-25%	5:26-50%	5:51-75%	5:76-100%	
*bcd*	0	0	2	0	2	0	0	4
*croc*	1	2	2	3	7	4	7	26
*D*	0	0	1	3	0	0	0	4
*Dfd*	0	0	0	4	5	4	1	14
*eve*	22	83	89	89	116	103	82	584
*fkh*	0	4	6	8	7	2	6	33
*ftz*	22	65	72	60	42	73	58	392
*gt*	1	24	27	28	22	16	10	128
*h*	0	2	2	3	3	1	0	11
*hb*	9	20	18	9	8	7	14	85
*hkb*	0	15	11	7	14	12	3	62
*kni*	9	8	10	10	9	16	11	73
*Kr*	1	11	23	9	14	15	4	77
*prd*	6	17	13	9	10	10	7	72
*rho*	0	2	8	16	3	13	10	52
*slp1*	1	2	6	6	13	29	12	69
*sna*	11	13	4	10	6	17	21	82
*tll*	0	0	0	0	0	4	2	6
*trn*	0	4	0	0	2	3	0	9
*tsh*	0	0	0	3	0	1	4	8
*twi*	2	4	7	11	11	7	4	46
*zen*	3	12	5	4	8	3	4	39
Total	88	288	306	292	302	340	260	1,876

Since each embryo was stained for two genes, the total given in each column is double the number of embryos in the release. The release contains some additional embryos for which the staging was ambiguous.

**Table 2:** The number of mutant PointClouds for *bcd*^12^, *gd*^7 ^and *Tl*^10B ^in Release 1 divided into the same developmental stages as in Table 1

Gene	Stage cohort							Total
		
	4d	5:0-3%	5:4-8%	5:9-25%	5:26-50%	5:51-75%	5:76-100%	
*bcd*^12^								
Mutant	1	0	2	5	10	10	11	39
WT-like	0	7	6	15	26	13	5	72
*gd*^7^								
Mutant	0	3	2	2	11	8	6	32
WT-like	0	3	1	5	13	4	0	26
*Tl*^10B^								
Mutant	0	4	5	4	9	1	2	25
WT-like	0	7	4	5	21	8	0	45

All embryos in *bcd*^12 ^and *Tl*^10B ^have been stained for *ftz *and *sna *mRNA expression. The embryos in *gd*^7 ^have been stained for *ftz *and either *sna *or *zen *expression. The number of PointClouds judged to be derived from homozygous mutant females (mutant) and heterozygous wild-type-like females (WT-like) are given. The release contains some additional embryos for which the staging was ambiguous.
